# Extirpation of a Tumour from the Antrum Maxillare, with Removal of the Superior Maxillary Bone, Including the Palate Plate

**Published:** 1847-01

**Authors:** C. Aston Key

**Affiliations:** Senior Surgeon to Guy’s Hospital


*Extirpatiou of a Tumour from the Antrum Maxillare, with removal of the superior Maxillary Bone, including the Palate Plate.* By C. Aston Key, F. R. C. S. E., Senior Surgeon to Guy's Hospital, Communicated by Sir Philip Crampton, Bart., F. R. S., &c.

*Merrion-square*, Sept. 20, 1846.

Dear Sir,--By the kind permission of Mr. Aston Key, I have the gratification of communicating to you the particulars of a case of tumour in the antrum maxillare, which was successfully removed by that excellent surgeon. The tumour was of great size, and must soon have proved fatal, as it had already pressed down the soft palate to such an extent as materially to interfere with deglutition. The aspect of the young woman was healthy; the tumour seemed to be of a firm nature; and there was no soft or fungoid-like protrusion through the nostril. These circumstances, no doubt determined Mr. Key to undertake the operation,

I remain, dear Sir, faithfully yours,

Philip Crampton.

To the Editor of Dub. Quarterly Journ. of Med. Science.

*St. Helen's-place*, Aug. 25, 1846.

My Dear Sir,—The operation of removing the superior maxillary bone in the young woman whom you saw at Guy's was done a fortnight ago, and she is now quite convalescent; so far the case, as you predicted, has proved quite satisfactory. The whole of the bony palate on one side, the nasal process, and the bone, with the exception of the floor of the orbit, were taken away, and exposed a large tumour of simple structure, which readily turned out from the back of the antrum. There was no difficulty in the operation, and the diseased mass was detached with ease from its bed. On examining the structure of the tumour we could discover no trace of malignant disease; it consisted of fibro-albuminous deposit, and, being of so benign a character, there is every prospect of its not returning. The incision in the cheek, which extended from the outer canthus of the eye to the angle of the mouth, healed by adhesion. I thought that you would like to know the result of the case.

I am, dear Sir, yours most truly,

C. Aston Key.

To Sir Philip Crampton, Bart.

*Dub. Quart. Journ. of Med. Science.*

